# Editorial: Small non-coding RNAs in Gram negative bacteria

**DOI:** 10.3389/fcimb.2024.1426124

**Published:** 2024-05-13

**Authors:** Bindu Subhadra, Meysam Sarshar

**Affiliations:** ^1^ College of Veterinary Medicine, Long Island University, Brookville, NY, United States; ^2^ Research Laboratories, Bambino Gesù Children’s Hospital-IRCCS, Rome, Italy

**Keywords:** small non-coding RNA (sRNA), Hfq, multidrug resistance, antibiotics, bronchiolitis, *Streptococcus pneumoniae*, outer membrane vesicles (OMVs)

Ongoing efforts to discover and characterize small non-coding RNAs (sncRNAs) in bacteria, often known as microRNA-size small RNAs (msRNAs) or more broadly as bacterial-derived small RNAs (bsRNAs), are deepening our knowledge of how they regulate post-transcriptional process. Although poorly described so far, they play an important role in controlling various biological functions of bacteria such as virulence, biofilm formation, antibiotic resistance, pathogenesis, adaptation to stress, and expression of outer membrane proteins. The objective of this Research Topic was to pool down the knowledge available so far in addition to attract promising new studies in sRNA regulatory network, which would enable forefront studies in the field of regulatory sRNAs to effectively tackle bacterial pathogens.

This Research Topic includes some original articles that explore how bacteria utilize sRNAs to survive under antibiotic stress conditions as well as their involvement in mediating differences in immune response in the case of respiratory syncytial virus versus rhinovirus bronchiolitis. It also includes valuable review articles that discuss Hfq interactions with sRNAs and how bacterial pathogens produce sRNAs encapsulated in outer membrane vesicles (OMVs). Through this process, sRNAs can be transferred into eukaryotic cells and other bacteria, highlighting their potential as therapeutic agents in the treatment of various diseases.

Kim et al. reported how bacteria switch from active aerobic respiration to anaerobic adaptation upon exposure to moderately effective first-generation antibiotics (Kim et al., 2024). The overuse and misuse of antibiotics has led to the emergence of multidrug resistant bacteria, and this situation has worsened with the overuse of antibiotics during Corona Virus Disease (COVID-19) pandemic (Andersson and Hughes, 2014; Rawson et al., 2020). The authors used a transcriptome analysis approach to understand the change in gene expression when the bacteria switch to anaerobic respiration (Kim et al., 2024). It has been noticed that the treatment of sublethal concentrations of antibiotics increased the expression of genes related to anaerobic respiration. In addition, the transition was dependent on the transcriptional regulators, AcrA (aerobic respiratory control) and FMR (fumarate and nitrate reduction) (Kim et al., 2024). It has been reported that the expression of these regulators is modulated by oxygen availability (Levanon et al., 2005). The authors in turn report that the antibiotic stress leads to specific reprogramming of non-coding RNAs including small RNAs FnrS and Tp2 (Kim et al., 2024). It was noticed that FnrS is involved in reducing reactive oxygen species (ROS) levels, thereby increasing cell survival. Overall, the study demonstrates how bacteria strive to maintain cellular homeostasis via sRNA-mediated gene regulation upon sublethal antibiotic exposure. As the authors pointed out, this study provides insights for developing novel antimicrobial compounds targeting sRNAs to combat multi-drug resistance.

Another valuable original article in our Research Topic demonstrates the role of bacterial sRNAs in mediating immune response in Bronchiolitis (Krohmaly et al., 2024). Bronchiolitis is a viral infection caused by many viruses including respiratory syncytial virus (RSV), rhinovirus (RV), and others (Marguet et al., 2009). It has been reported that bronchiolitis caused by RSV is majorly associated with *Streptococcus pneumoniae*, while bronchiolitis caused by RV is frequently associated with *Haemophilus influenzae* (Hasegawa et al., 2018; Stewart et al., 2018). In this study, the authors identified many novel sRNAs from different bacterial species and studied their influence on immune response during bronchiolitis (Krohmaly et al., 2024). Through RNA-Seq database, several bacterial sRNAs were found to be associated with RSV and RV-only bronchiolitis in human nasal. They found that some bacterial sRNAs were differently expressed in infants with RSV compare to RV-only bronchiolitis from the MARC-35 cohort. They found that the sRNAs associated with RSV-only bronchiolitis may relatively activate the IL-6 and IL-8 pathways and relatively inhibit the IL-17A pathway, compared to those associated with RV-only bronchiolitis (Krohmaly et al., 2024). This is the first study to report that bacterial sRNAs may be contributing to inflammation differences seen in RSV- and RV-only bronchiolitis.

Nowadays, it is known that production and regulation of bacterial sRNAs that are involved in facilitating sRNA-mRNA base-pairing is coordinated through several components, including other sRNAs, mRNAs, and sRNA-binding proteins (sRBPs). Among several sRBPs (e.g., Hfq, ProQ, and CsrA), Hfq is the most extensively studied chaperon that protects sRNAs from degradation and aids in their binding to mRNA. Watkins and Arya have written an interesting review on how the structures of Hfq have defined the difference in interactions of the Gram-negative and Gram-positive homologues with RNA (Watkins and Arya, 2023). While it appears that Hfq is a vital virulence factor in Gram-negative bacteria, it remains unclear how this chaperone is involved in mediating sRNA-mRNA interactions, a function that is worth exploring.

Another interesting review paper published by Ajam-Hosseini et al. discussed bacterial OMVs that can serve as vehicles for the delivery of sRNAs to target cells (Ajam-Hosseini et al., 2024). It has been shown that sRNAs encapsulated in OMVs can regulate gene expression in recipient cells, leading to changes in cellular behavior and function. Additionally, targeting sRNAs involved in bacterial virulence or antibiotic resistance has the potential to disrupt the pathogenicity of bacteria and improve the effectiveness of antibiotic treatment. This suggests that OMV-encapsulated sRNAs could be used as potential therapeutic strategy in treating various bacterial diseases.

In summary, the current Research Topic highlights the importance of regulating bacterial sRNAs in shielding bacterial lifestyle under stress conditions and in modulating the immune response. It also provides valuable insights into the clinical significance of Gram-negative bacterial sRNAs in biomedical applications. Despite its importance, detailed studies on the expression patterns of bacterial sRNAs are scarce. Therefore, we believe that further research could enhance our understating of their role as a versatile toolkit for bacterial adaptation to the host environment. Such studies could also reveal their potential utility as novel therapeutics, including their use in natural or synthetic OMVs.

We would like to express our gratitude to the authors and reviewers for their valuable contributions to this Research Topic. We hope that this collection of reviews, and original articles will be helpful for clinicians, researchers, and students seeking for information about sRNAs in bacterial virulence and communication.

## Author contributions

BS: Conceptualization, Supervision, Validation, Visualization, Writing – original draft, Writing – review & editing. MS: Conceptualization, Supervision, Validation, Visualization, Writing – original draft, Writing – review & editing.
